# Lights and Dots toward Therapy—Carbon-Based Quantum Dots as New Agents for Photodynamic Therapy

**DOI:** 10.3390/pharmaceutics15041170

**Published:** 2023-04-06

**Authors:** Svetlana Jovanović, Zoran Marković, Milica Budimir, Jovana Prekodravac, Danica Zmejkoski, Dejan Kepić, Aurelio Bonasera, Biljana Todorović Marković

**Affiliations:** 1 Vinča Institute of Nuclear Sciences—National Institute of the Republic of Serbia, University of Belgrade, P.O. Box 522, 11000 Belgrade, Serbia; 2 Palermo Research Unit, Department of Physics and Chemistry—Emilio Segrè, University of Palermo and Consorzio Interuniversitario Nazionale per la Scienza e Tecnologia dei Materiali (INSTM), 90128 Palermo, Italy

**Keywords:** graphene quantum dots, carbon quantum dots, carbon nanodots, carbonized polymer dots, phototoxicity

## Abstract

The large number of deaths induced by carcinoma and infections indicates that the need for new, better, targeted therapy is higher than ever. Apart from classical treatments and medication, photodynamic therapy (PDT) is one of the possible approaches to cure these clinical conditions. This strategy offers several advantages, such as lower toxicity, selective treatment, faster recovery time, avoidance of systemic toxic effects, and others. Unfortunately, there is a small number of agents that are approved for usage in clinical PDT. Novel, efficient, biocompatible PDT agents are, thus, highly desired. One of the most promising candidates is represented by the broad family of carbon-based quantum dots, such as graphene quantum dots (GQDs), carbon quantum dots (CQDs), carbon nanodots (CNDs), and carbonized polymer dots (CPDs). In this review paper, these new smart nanomaterials are discussed as potential PDT agents, detailing their toxicity in the dark, and when they are exposed to light, as well as their effects on carcinoma and bacterial cells. The photoinduced effects of carbon-based quantum dots on bacteria and viruses are particularly interesting, since dots usually generate several highly toxic reactive oxygen species under blue light. These species are acting as bombs on pathogen cells, causing various devastating and toxic effects on those targets.

## 1. Introduction to Photodynamic Therapy

The number of patients with carcinoma and infectious diseases increases, no matter the development of diagnostics tools and medication. With 10 million deaths in 2020 worldwide, cancer is a leading threat [1]. Thus, there is a tremendous need for new, efficient, minimally invasive, and affordable therapy. In addition to tumors, a large number of extremely dangerous microbes (bacteria and viruses) constantly increase the death rate and reduce patient life quality. Antibiotics and antiviral agents are major weapons in the fight against infections. However, the development of microbial resistance to drugs over time is a global issue. Could visible light-triggered photoactive nanoparticles be a salvation for these global challenges? 

New approaches for visualizing tumor tissues were developed in the 1940s and 1960s [2,3,4], and porphyrins were studied along with sunlight-activated eosin [5]. Lipson used hematoporphyrin as a tumor probe [6]. In the 1970s, fluorescein diacetate and porphyrins were used to detect and eradicate tumors [7,8]. This is how photodynamic therapy (PDT) was born.

What is the basis of photodynamic therapy? What is a photosensitizer? 

**Figure 1:**
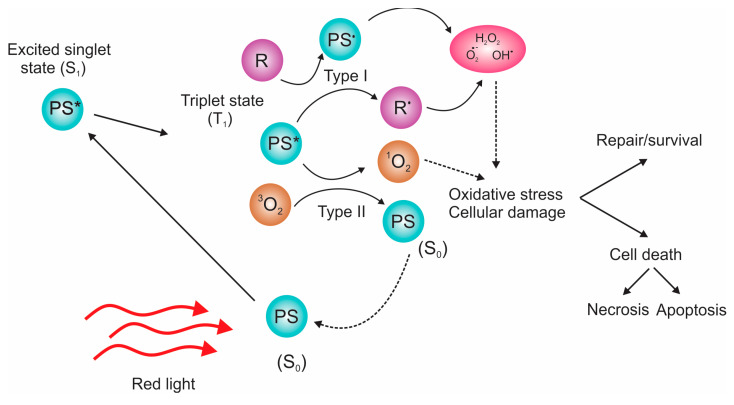
The mechanism of PDT: when PSs are irradiated by light, they transfer from singlet ground state (S_0_) to excited singlet state (S_1_), followed by intersystem crossing to excited triplet state (T_1_). ROS are generated by transferring energy from T_1_ via Type I and Type II reactions.

The three principal components of PDT are the photosensitizer (PS), light, and oxygen molecules, as depicted in Figure 1 [9]. PSs are molecules or materials that can be activated by light, are able to transfer energy to oxygen molecules, and generate cytotoxic reactive oxygen species (ROS) (Figure 1) [10]. PSs are required to have the following properties: (I) absorb the light effectively at a certain wavelength, (ii) have appropriate energy at the triplet state to provide sufficient energy for transfer to the ground state, (iii) possess an appropriate quantum yield, (iv) possess a long lifetime at the triplet state, and (v) have appropriate and high photostability [11]. 

The advantages of cancer PDT are the following [12,13]:

- The PSs tend to build up in abnormal cells, and the application is focused strictly on them; that is why the damage to healthy cells is limited;
- No longterm side effects, and possibility for repeated treatments at the same position;
- Less invasive than surgery, and shorter recovery time;
- Immune activation ability;
- Usually costs less than other cancer treatments;
- Does not cause scarring, and is appropriate for treating skin or eye cancers.

However, because this kind of therapy has limitations, such as damage to normal cells and low depth of light penetration in tissue [14], it is not appropriate for cancers that have already affected widespread portions of the patient’s body, and some PSs leave people very sensitive to light for several days. The depth of light penetration through tissues is affected by the wavelength of the incident light, its intensity, coherence, polarization, tissue hydration, the presence of pigments, fibrotic structures, and composition [15]. The depth that UV light can reach is only 50 to 150 μm; 1 mm for blue (425–475 nm), 2 mm for green (495–550 nm), and 10 mm for red (650–940 nm) [16]. Although the NIR range is most suitable, considering tissue depth penetration, PSs are often inefficient in ROS production under this light. Thus, new approaches are being developed, such as X-ray-induced ROS production [17]. When nanoparticles are exposed to X-rays, they emit scintillation or persistent luminescence, leading to PS activation and ROS production. Instead of irradiation light, sonodynamic therapy uses ultrasound to excite sensitizers, such as copper–cysteamine, and to induce ROS production [18]. Another approach to resolve the issue of depth penetration is microwave irradiation [19]. Recently, specific PSs were able to produce ROS when induced by microwave at 10 W (2450 MHz), which is a base for a new microwave dynamic therapy (MWDT).

Apart from cancer, PDT could be used for the treatment of different types of infections. Antimicrobial PDT is based on new types of PSs which are able to produce ROS, that kill bacteria unselectively via oxidative stress [20]. These PSs are not cytotoxic without light [21,22,23]. In addition to the rapid effect on pathogens, the obstacles with PSs are related to the synthesis and isolation methods, and modifications to be easily uptaken by microbes [24]. The most challenging issue is fighting the biofilms formed by pathogens as their protection against antibiotics, which leads to even higher drug resistance [25]. 

**Figure 2:**
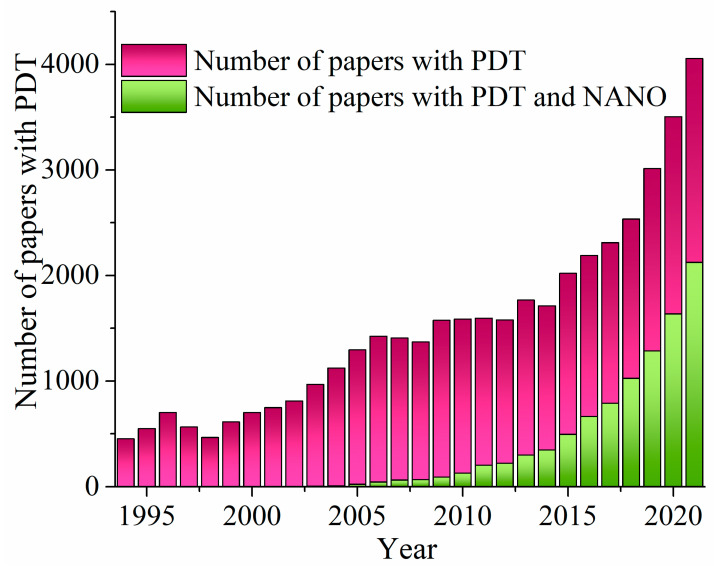
The number of published papers with “photodynamic therapy” as a keyword (violet) per year, and the number of papers published with the keywords “photodynamic therapy” and “nano*”, according to the Scopus database, on 1 October 2022.

In recent decades, PDT has attracted significant scientific attention (Figure 2, violet bars). Within these results, research that involved the application of nanomaterials in PDT has undoubtedly received a great deal of interest over the past several years (Figure 2, green bars). 

Thus, carbon-based nanomaterials, particularly dots, are stepping into the spotlight as new PDT agents due to their ability to produce ROS after exposure to light, their biocompatibility, good solubility in water, stability in physiological fluids, resistivity to photobleaching, chemical inertness, and their fast elimination from organisms.

This review will summarize all the properties of several types of carbon quantum dots that are suitable as PSs. Initially, their structural, optical, and chemical properties will be reported, as well as their potential for ROS generation, including different parameters that influence the ability of ROS production, such as structural modification by doping, gamma rays, or even plasma treatment. We have summarized the available literature data regarding the effects on tumor cells, different bacteria strains, and cytotoxicity.

## 2. Structure and Properties of Carbon-Based Dots

Carbon dots (CDs) are considered a kind of 0D carbon-dominated nanomaterial, with a size of less than 20 nm, consisting of a sp^2^/sp^3^ carbon skeleton and abundant functional groups/polymer chains. A large amount of surface groups/polymer chains gives rise to their excellent solubility in water or organic solvents, and makes them convenient for composing with other polymer materials without phase separation.

**Figure 3:**
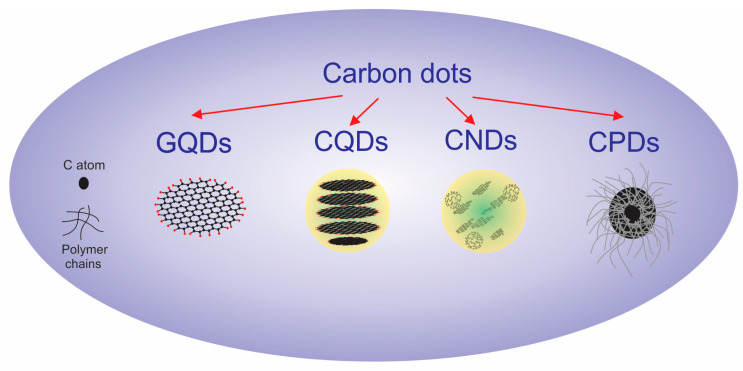
Types of carbon dots: GQDs, CQDs, CNDs, and CPDs.

CDs are reported as singlet oxygen generators, resistive to light decomposition, photobleaching, photoblinking, high photoluminescence quantum yield, low toxicity, low production costs, and excellent biocompatibility. CDs include graphene quantum dots (GQDs), carbon quantum dots (CQDs), carbon nanodots (CNDs), and carbonized polymer dots (CPDs), which are classified according to the specific carbon core structure, surface groups, and properties (Figure 3 and Table 1) [26,27,28,29,30,31].

Taking into account those keywords, CDs can be grouped according to their structure, starting material for production, crystallinity, polymer content, synthesis, and main properties, as presented in Table 1.

### 2.1. Graphene Quantum Dots, Structure, and Properties

Graphene quantum dots were produced for the first time in 2008 by Ponomarenko et al. [32]. Later, graphene on Si support was tailored with electron-beam lithography [33]. Subsequently, a large number of synthetic approaches were developed [34,35,36,37,38,39,40,41,42,43,44,45,46]. 

**Figure 4:**
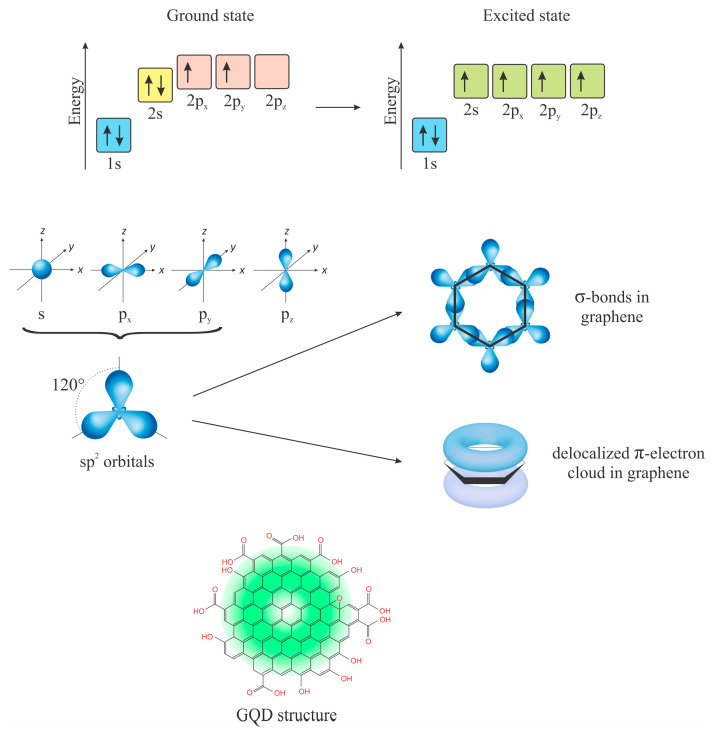
Electronic configuration of C atom, hybridization, and electronic cloud in benzene rings, and the electronic cloud in GQDs.

GQDs mainly have sp^2^ carbon atoms organized in honeycomb structures (Figure 4). Due to the same energy and small distance between C atoms, a unique electronic cloud is created. Here, electrons are traveling freely as long as the cloud is continual (Figure 4, GQD green cloud). If the cloud is disrupted, electron movement is limited. The size of GQDs is usually below 100 nm, and they show all the characteristics of quantum dots, but their lateral size is not correlated with the expected energy band gap. Why is that? 

The size of GQDs that is observed by microscopes is not the same as the size of the π-cloud. Instead of a unique π-cloud, there are discontinuities. Thus, the actual size of the semiconducting area is largely different than the physical size of the dots. This condition is because the π-cloud in GQDs is disrupted into small-sized aromatic sp^2^ domains, due to the presence of holes in the graphene, or functional groups. 

Graphene is responsible for the photoluminescence (PL) of GQDs. Pan et al. first detected this feature in 2010 [47], and assigned it to free zigzag sites with a carbene-like triplet ground state (σ^1^π^1^) in small objects (ca. 9.6 nm). Herein, the actual size of graphene sheets was not considered. Graphene is a 0-band gap semiconductor [48]. The quantum confinement effect occurs when the object’s diameter is smaller than its exciton Bohr radius [49,50,51]. While classical semiconducting QDs are built from large molecular weight atoms, GQDs are made of lightweight atoms with a small dielectric constant. Due to strong carrier–carrier interactions, and the formation of new electronic states, the band gap of GQDs is much larger compared to conventional QDs with the same lateral size. Most of the GQDs show yellow, blue, or green PL thanks to a large band gap [37,52,53,54,55,56]. While the graphene core dictates the intrinsic emission through the quantum confinement effect, functional groups govern the surface state emission. 

The three most important factors for PL behavior are:

- The size of the core (graphene);
- Edge configuration (zigzag or armchair);
- The physicochemical nature of functional groups.

The structural effects affecting PL emissions are:

- Carboxyl and amide groups mainly cause green emissions;
- Hydroxyl groups contribute to blue emissions [57];
- Red emissions depend on the sp^2^-conjugated size, and surface states control emission [58];
- Amine groups are electron-donating, increasing the electron density, and lowering the band gap [59];
- OH groups lead to various levels of disruption of the conjugated π-system, changing the dots’ structural flexibility, and making them more rigid [60].

Table 2 summarizes the GQDs with different structural and morphological properties. As can be seen, the PL quantum yield varies largely, from 1.1 to 99.8%, as well as the color of the PL emission, from violet to near-infrared. 

**Figure 5:**
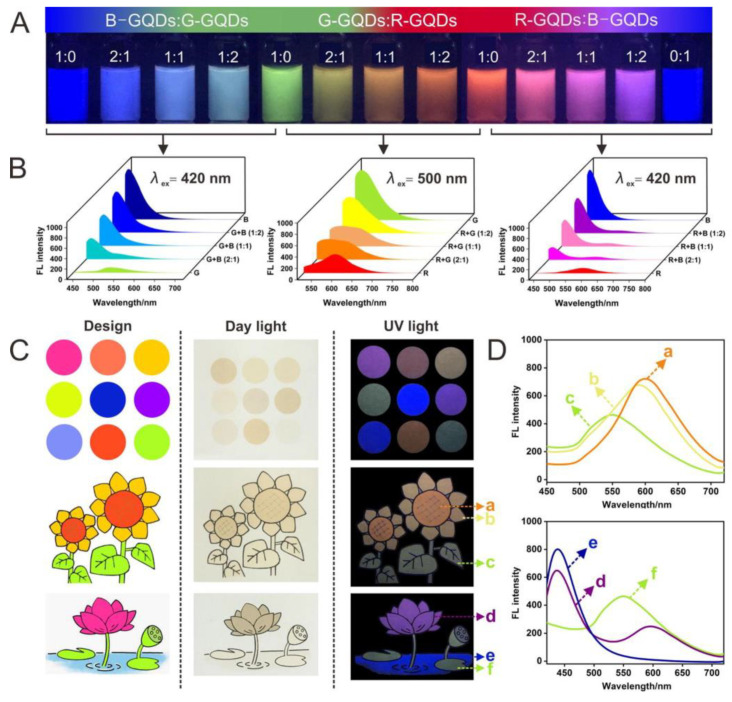
(**A**) Color range of GQDs mixtures, (**B**) emission PL spectra of selected mixtures, (**C**) printed GQDs images under daily and UV lights and (**D**) emission PL spectra of region that was indicated by arrows. Reprinted from *Journal of Colloid and Interface Science*, 579, Jingwen Zhao, Yanyan Zheng, Youyou Pang, Jie Chen, Zheye Zhang, Fengna Xi, and Peng Chen, *Graphene quantum dots as full-color and stimulus-responsive fluorescence ink for information encryption*, 307, copyright (2022), with permission from Elsevier [78].

As an example of the ability of GQDs to emit light of different colors under 365 nm excitation, Figure 5 is presented [78]. Here, a wide color range (Figure 5A) was achieved by mixing various GQDs, while emission spectra showed a significant color shift (Figure 5B). These dots were used to produce inks by dispersing them in ethanol, with the addition of glycerol, which increased ink viscosity, and dots were printed (Figure 5C).

GQDs possess various oxygen-containing functional groups in their structure: carboxyl, hydroxyl, carbonyl, epoxy, ethoxy, lactone, or ester. A low-intensity PL is a nonradiative electronic relaxation that is a result of thermal decay [81]. When the fast electrons–holes recombination occurs, the density of charge carriers is lowered, leading to a lower number of particles available for photon conversion. One of the strategies for increasing PLQY is to functionalize GQDs in a manner that blocks vibration relaxation. Thus, several approaches are developed: surface passivation by functionalization of GQDs with polymers [82], amino functionalization [83], removal of O functional groups by chemical reduction [84], or gamma irradiation [27,85,86]. However, the specific role that each oxygen-containing group plays in the loss of nonradiative recombination of GQDs is still unclear.

Oxygen and other functional groups are more or less polar, and they are responsible for the solubility of GQDs in water and polar organic solvents, such as methanol, ethanol, dimethylsulfoxide, etc. [87,88,89,90]; however, certain modification can change their solubility, and increase solubility in ethers, methanol, n-hexane, heptane, xylene, dichloromethane, and toluene [91]. 

Due to their rich chemistry and various approaches for their structural modification, their optical and physicochemical properties can be easily tuned, which makes them highly attractive materials at the present time. 

### 2.2. Carbon Quantum Dots

**Figure 6:**
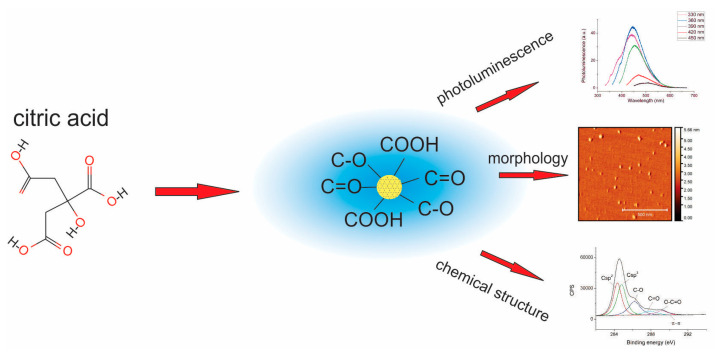
Chemical structure, morphology, and photoluminescence of CQDs synthesized from hydroquinone in the hydrothermal reactor at 180 °C for 12 h. Detailed information can be found in ref. [29].

Carbon quantum dots are a subgroup of carbon dots with a quasi-spherical shape, a typical lateral dimension below 10 nm, and an average height of about 1–2 nm [92]. Their carbon honeycomb network is predominantly composed of amorphous and crystalline cores, with either graphitic or turbostratic carbon or graphene and graphene oxide sheets mixed by sp^3^ carbon bonds (Figure 6). They are characterized by good chemical and thermal stability, tuneable photoluminescence and optical band gap, resistance to photobleaching, and low dark cytotoxicity [93]. 

There are two general approaches to produce CQDs: bottom-up and top-down [92]. In general, the bottom-up method implies the usage of a hydrothermal or microwave reactor, as well as solvothermal treatment or pyrolysis [92,94,95,96]. CQD synthesis in the hydrothermal reactor can be considered a green, eco-friendly, nontoxic procedure in which different materials can be used as starting precursors to prepare CQDs: citric acid, carbon hydrates or glucose, chitosan, banana, cabbage, etc. [97,98,99,100,101,102]. As starting carbon precursors, glucose, sucrose, succinic acid, catechol, resorcinol, and hydroquinone are used, whereas polyethylene glycol, NH_4_OH, and tris(2-aminoethyl)amine are used as reaction media during synthesis in the microwave reactor [96,103,104,105]. During the synthesis of CQDs by solvothermal treatment, carbon precursors are heated in high boiling point organic solvents, followed by filtration and dialysis [92,106].

The other method, top-down, implies the usage of graphene, carbon nanotubes, graphite, and graphene oxide, which activate carbon to synthesize CQDs [92,96].

Due to the presence of epoxy, carbonyl, and carboxyl groups on the surface of CQDs, these nanoparticles possess very good dispersibility in water/organic solvents [107]. However, they can be obtained in the hydrophobic form as well. Both hydrophilic and hydrophobic CQDs were produced simultaneously by varying the H_3_PO_4_/ethanol molar ratios within 0–1.72, while hydrophilic or hydrophobic CQDs are the sole product obtained from H_3_PO_4_–BmimPF_6_ or BmimPF_6_-only systems [108]. Hydrophobic CQDs were deposited as a thin film on glass, and SiO_2_ showed a very high ability to produce singlet oxygen [109]. These CQDs are prepared from polyoxyethylene–polyoxypropylene–polyoxyethylene Pluronic 68. One of the problems with depositing continuous and uniform hydrophilic CQDs thin films is their water solubility, which prevents the formation of the Langmuir layer at the water subphase [30]. Thus, CQDs dispersed in organic solvents should be prepared. Pan et al. prepared hydrophobic CQDs through the formation of amide linkage (–NHCO–) from –COOH covalently linking with dodecylamine (DDA) in a thermal reaction process; the carbon nanoparticles became hydrophobic and were automatically transferred into the toluene phase [110]. Mitra et al. prepared hydrophobic CQDs from polyoxyethylene–polyoxypropylene–polyoxyethylene Pluronic 68 in a microwave reactor, and they showed very good dispersibility in different organic solvents, such as acetone, ethanol, chloroform, toluene, THF, NMP, hexane, cyclohexane, DMF, and acetonitrile [111]. Hydrophobic CQDs can be encapsulated into polymers, such as polyurethane or polydimethylsiloxane, by the swelling-encapsulation-shrink method. These nanocomposites showed very good potential to generate singlet oxygen [112,113]. Gamma rays induced structural modifications of CQDs/polyurethane composites, which contributed to their increased ROS production compared to nonirradiated samples [114]. Plasma-treated hydrophobic carbon quantum dots/polydimethylsiloxane nanocomposites enhanced the pro-oxidant properties of these nanocomposites [115]. 

One of the very important properties of CQDs, that can be tuned in different ways, is their ability to emit light in various regions of the electromagnetic spectrum. Although there are several theories related to the photoluminescence of CQDs, the common features of all the theories are: the quantum confinement effect, or conjugated π-domains and surface defects, i.e., surface states are responsible for the photoluminescence of CQDs [116]. Molecular PL is imprinted by fluorescent molecules attached to the surface and/or interior of the CDs [117]. Photoluminescence emission spectra of CQDs are dependent on the excitation wavelength, and there is an up- or downshift of emission PL spectra compared to excitation [118]. One study showed that, in the case of S,N-codoped CD, the center of the emission band was correlated with the dot diameter, and the size effect was the essential factor for the fluorescence of these dots [119].

Depending on starting precursors, solvent, and synthetic procedure, the photoluminescence of CQDs can be tuned. Thus, the functionalization of CQDs by amino groups (–NH_2_ groups) induces a red-shift of photoluminescence, due to charge transfer from amino groups to the carbon honeycomb core [120]. Additionally, –NH_2_ groups enhance the affinity of CQDs to biological structures, whereas the incorporation of nitrogen atoms in the honeycomb structure of CQDs contributes to the reduction of photobleaching [121]. The incorporation of CQDs in PAM leads to phosphorescence, due to the formation of hydrogen bonds between dots and polymer [122]. In this way, the T1 state of CQDs was locked and induced a larger number of triplet excitons, and prevented nonradiative emission. Furthermore, the increase of N content prevents T1 from nonradiative inactivation. Blue photoluminescence of CQDs is a feature of the quantum size effect and zigzag edges, whereas the red-shifted photoluminescence is predominantly due to surface defects presented on the basal plane and edges of the sp^2^ domain inside the sp^3^ matrix, and the increased size of the aromatic π-conjugated domains [123].

### 2.3. Doped Carbon Quantum Dots

CQDs show certain drawbacks, such as poor quantum yield compared to inorganic semiconductor QDs, insufficient utilization of visible light, and monochromatic fluorescence, which hinder their broad applications [124]. Short penetration depth in tissues limits their full potential as PSs [125]. Hence, it is important to modify their fluorescence properties, to expand the spectrum of their applications. 

Two successful strategies for the improvement of CQDs’ photoluminescence performance are surface functionalization and heteroatom doping. The introduction of surface functional groups, either via covalent or noncovalent modification, greatly affects the PL quantum yields of CQDs, their quenching capability, and the PL color [126]. All of the benefits of the surface functionalization of CQDs, and the following critical challenges, were discussed in detail elsewhere [121,127,128]. On the other hand, heteroatom doping introduces heteroatoms, either metal ions or nonmetal atoms, in the structure of CQDs, which is primarily composed of C, O, and H. The introduced heteroatom changes the electronic structure of CQDs, affecting their energy gap. CQDs exhibit σ→σ*, σ→π*, π→π*, n→π*, and n→σ* electronic transitions that are related to their optical properties, precisely, their PL, and absorption [129]. It is estimated that heteroatoms in CQDs have an impact on the interactions between π and n states in CQDs, due to the extent of overlap of the orbitals and the heteroatom’s ability to accept or donate electrons [130]. 

**Figure 7:**
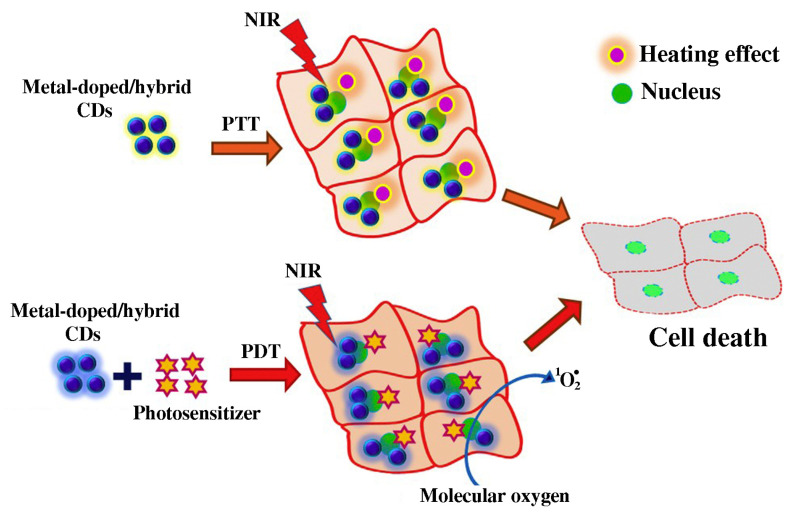
Photodynamic and photothermal therapy induced by metal-doped/hybrid CDs. Reprinted from *Journal of Controlled Release*, 330, Neeraj Tejwan, Adesh K. Saini, Anirudh Sharma, Th. Abhishek Singh, Nitin Kumar, and Joydeep Das, *Metal-doped and hybrid carbon dots: A comprehensive review on their synthesis and biomedical applications*, pages 132–150, copyright (2021), with permission from Elsevier [138].

The introduction of metal ions into CQDs significantly improves their physicochemical properties, particularly their photothermal and photodynamic effects (Figure 7) [131]. The presence of unoccupied orbitals in metal ions, and their electron donor abilities, changes the charge density and charge transition forms among metal ions and the carbon lattice of CQDs upon doping [130]. As a result of the modified electronic structure of CQDs, their energy gap between the highest occupied molecular orbital (HOMO) and lowest unoccupied molecular orbital (LUMO) also changes, which, consequently, has an impact on their optical properties. Compared to nondoped CQDs, metal-doped CQDs have superior PL and greater quantum yield, while improved catalytic performances and relaxation properties have also been found [132]. Doped CQDs usually show typical absorption peaks in the UV region, attributed to the π–π* and n–π* transitions stemming from the carbon framework [133,134,135]. Due to the presence of a metallic dopant, a metallic ion–carbon framework charge transfer is generated, which causes the enhanced absorbance of doped CQDs in the visible region [136,137]. The emitted PL of metal-doped CQDs, under an excitation wavelength of 365 nm, range from ultraviolet [138], blue [134,135,137,139,140], blue-green [141], green [142], and yellow [133,143,144], among others. The majority of doped CQDs shows excitation-dependent photoluminescence [134,140,145]. Metal doping of CQDs causes a red-shift of the PL emission peak [133,144]. The improvement in PLQY upon doping is ascribed to the emerged emission energy traps, which enhance electron–hole recombination [146], or the presence of the surface plasmonic resonance (SPR) effect in metal nanoparticles [147]. A change in the fluorescence of Mn-doped CQDs upon solvent polarity change was demonstrated [148]. 

On the other hand, since metals have larger radii than carbon, doping with metals might result in the nonuniform distribution of dopants in CQDs [149]. Another concern is the potential toxicity of metal ions, which could greatly hinder metal-doped CQD applications in a biological context [150]. Gd-doped CQDs intravenously injected into mice did not induce tissue pathological damage [151]. Additionally, various metal-doped CQDs showed low toxicity on HO-8910 cells [145], L929 cells [152], human epidermoid cancer cells [141], C6 cells [142], and HepG2 cells [151].

**Figure 8:**
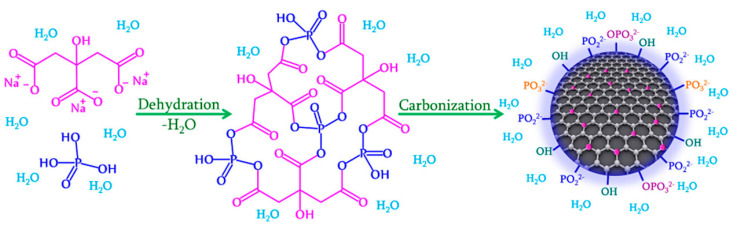
Schematic representation of the synthesis of P-doped CQDs from trisodium citrate and phosphoric acid. Reprinted from *ACS Omega*, 5, G. Kalaiyarasan, J. Joseph, and P. Kumar, *Phosphorus-Doped Carbon Quantum Dots as Fluorometric Probes for Iron Detection*, pages 22278–22288, copyright (2020) [155]. This work is licensed under the Creative Commons public use license, and further permissions related to the excerpted material should be directed to the ACS.

The doping of CQDs with nonmetals usually implies elements such as N, S, P, F, Se, and B (Figure 8) [109,153,154,155,156,157]. The resemblance between their size and the size of C is reflected in the uniform doping of CQDs. The dopant changes the surface structure of the CQD carbon framework, which results in the creation of new excited energy levels. The electronegativity of the dopant also plays an important role in the photoluminescent properties of doped CQDs. 

CQDs doped with highly electronegative elements, such as N and S, create blue-shifted PL emissions, while the elements with lower electronegativity (P and B) create red-shifted emissions [149,158]. The size of a dopant also affects the properties of the doped CQDs. For example, N atoms have comparable sizes to C atoms. This, combined with the presence of five valence electrons of nitrogen, could lead to an increase in the surface-state defects of N-doped CQDs, which consequently increases their PLQY [159]. Nitrogen in N-doped CQDs can be present as pyridinic, pyrrolic, and graphitic, and can be located either at the edge of, or inside, CQDs [160,161]. The atomic radius of sulfur is bigger than the radius of carbon, and the existing mismatch between the outermost orbitals of S and C creates an uneven distribution of the spin density, which is reflected in the properties of S-doped CQDs [162]. Another dopant with an atomic radius larger than carbon is phosphorus. Phosphorus behaves as an n-type donor, and P-doping of CQDs improves their photoelectric properties. It was found that P-doping effectively modifies the electronic properties of CQDs by generating a large number of active sites, which enhances their fluorescence properties and stability [163]. While all the aforementioned dopants (N, S, and P) increase the electron concentration of CQDs upon doping, boron is deficient in electrons, which creates defects in the energy states of CQDs, and further leads to emissions from the surface defects of the CQDs [164].

The most-used method to prepare doped CQDs is the solvothermal or hydrothermal method [165,166,167,168,169]. Another method to obtain metal-doped CQDs is the reduction method, where CQDs act as reducing agents [170,171,172]. This method is simple and cost-effective, since it does not involve high temperatures. The microwave (MW) irradiation method uses microwaves to achieve high temperatures for a short amount of time [173,174]. The MW method is more economical. In the sonochemical method, localized high temperatures and pressures, essential for the preparation of doped CQDs, are achieved by ultrasound waves [175]. Apart from these, other CQDs preparation methods, such as the pulsed laser irradiation method [176], the seed-mediated method [177], and the carbonization method, were also reported [178].

## 3. ROS Production from Carbon-Based Dots

Reactive oxygen species generation depends on the number and spatial distribution of aromatic carbon islands, the CD solvent, and the type of polymer used for nanocomposite design, while ROS deactivation primarily depends on the type, number, and spatial distribution of functional groups. ROS generation predominantly proceeds through the interaction of aromatic electrons of CDs that collide with oxygen electrons. As a result, the internal energy of the oxygen molecule is increased, and the spin is changed. CDs have similar properties to the family of expensive carbon molecules (fullerene, porphyrins, etc.) that are highly potent photosensitizers [179,180,181,182]. 

One of the most abundant functional groups of water-soluble CDs is the hydroxyl group. Based on the lifetime of singlet oxygen in a variety of solvents, Hurst and Schuster calculated the singlet oxygen-quenching rate constants for different X–Y bonds, and found that the –O–H bond should quench singlet oxygen most efficiently (rate constant 10^3^ L M^−1^ s^−1^) [183]. The ^1^O_2_-scavenging capacity of alcohols exponentially increases with the number of –OH groups (rate constants 10^3^, 10^6^, and 10^9^ L M^−1^ s^−1^ for some monohydroxy-, dihydroxy- and trihydroxy-alcohols, respectively). Similarly, the ^1^O_2_-deactivating capacity of malonic acid CH_2_(COOH)_2_ [rate constant 10^4^ L M^−1^ s^−1^] is one order of magnitude higher than that of acetic acid (CH_3_COOH, rate constant 10^3^ L M^−1^ s^−1^), which demonstrates the quenching properties of the carboxylic group [184]. We, therefore, propose that such an unusually high rate of increase in singlet oxygen-quenching capacity might also occur with increasing CDs functionalization, thus endowing the highly functionalized CDs with the excellent ability of ^1^O_2_ deactivation.

- Electrochemical cutting of graphite electrodes leads to the formation of GQDs functionalized with –OH groups (formed on the cathode), and GQDs functionalized with –CH groups (formed on the anode) [26]. The first type is water soluble, while the second one is soluble in acetone and toluene. Even though sp^2^ aromatic content is relatively large, –OH and –COOH groups quench singlet oxygen in aqueous solutions very efficiently. Acetone-soluble, and especially toluene-soluble GQDs, produce a significantly higher amount of singlet oxygen [unpublished data].
- CQDs are soluble in aqueous solvents, and have many functional groups that quench singlet oxygen. Some types of CQDs are excellent antioxidants [185,186,187,188,189,190,191]. As source material, fruits and vegetables with known antioxidant properties are commonly used. In Table 3, the DPPH scavenging activity for a variety of CQDs is presented:

**Table 3:** Values of DPPH scavenging activities for selected CDs.

Title	Source Material	Scavenging Activity (%)
CQD [185]	Tannic acid	84.5
CQD [186]	Ananas	23.3
GQD [188]	Pyrene	80
Cl-CQD [189]	Citric acid, urea, NaCl	88
CQD [190]	Tomato	63.8
CQD [191]	Pomelo	56
T-CQD [192]	Thumbai	89
CQD [193]	Taurine	82.5
S-CQD [194]	Turmeric and ammonium persulfate	79.5
CD [195]	Carica papaya leaves	86
CD [196]	Beta vulgaris	94.5
CQD [197]	Citrus clementina peel	81.4
TCD [198]	Green tea	75
rcCQD [199]	Red cabbage	61
N-CD [200]	Black soya	93.8
N,S-CD [201]	Pomelo and sulfamic acid	82

- The CNDs possess rather similar ROS generation and quenching properties to CQDs.
- The polymer content of CPDs provides excellent solubility in solvents, as well as a low quenching ability of singlet oxygen [112,202]. Quenching is especially low if copolymers are used for synthesis, with hydrophobic parts rich in methyl groups [109]. The architecture of the carbon core and the size of the π-conjugated domain are crucial for singlet oxygen generation.

Carbonization is very important for the formation and properties of CPDs [203,204]. Usually, the carbonization degree increases with the increasing of thermal treatment temperature and reaction time. The transition from the molecular state to a solid carbon core significantly affects the capacity of singlet oxygen generation.

The methods for the detection of singlet oxygen produced by CDs are classified as direct and indirect. The most reliable direct method measures the luminescence of singlet oxygen at 1270 nm. Indirect methods are based on electron paramagnetic resonance, visible photoluminescence, or UV-Vis. These methods use probes (TMP, ABDA, DBPF) that react with singlet oxygen, and the optical response of the products is measured. Since the majority of CDs are luminescent in the visible spectrum, the detection of singlet oxygen at 1270 nm is straightforward. Electron paramagnetic resonance (EPR) measurement of (2,2,6,6-tetramethylpiperidin-1-yl)oxyl (TEMPO) is also easy, since carbon dots commonly lack unpaired electrons. The measurement of probes by visible photoluminescence or UV-Vis can be problematic, due to the overlapping of spectra of the CDs and photolabile probes. Table 4 summarizes the data on the quantum yield of singlet oxygen of CDs, measured by different groups.

## 4. Anticancer PDT with Carbon-Based Dots

### 4.1. Photodynamic Therapy with GQDs

A pioneering study in the field of GQDs application as a PS was conducted by our group [216]. For the first time, human glioma cells U251 were treated with GQDs. U251 cells were treated with 200 μg mL^−1^ GQDs and blue light (470 nm), and a cell viability of approximately 40% was observed. Our study proved that GQDs’ phototoxicity was related to the production of ROS. 

**Figure 9:**
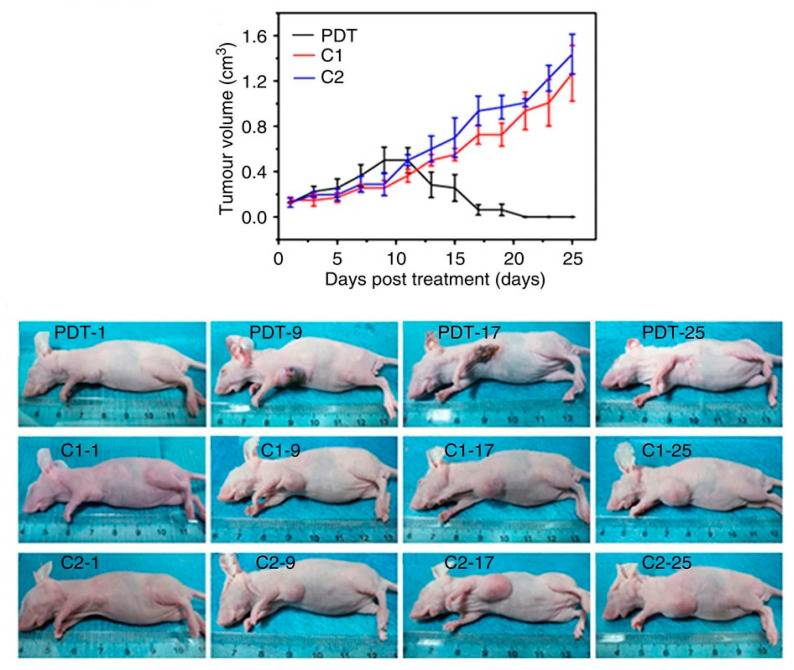
In vivo PDT. Time-dependent tumor growth curves (*n* = 5) after different treatments. *p* < 0.05 for each group. Photographs of mice after various treatments on the 1st, 9th, 17th, and 25th day. (PDT: GQDs and light irradiation; C1: GQDs only; C2: light irradiation only.) Adapted from *Nature Communications*, 5, by Jiechao Ge, Minhuan Lan, Bingjiang Zhou, Weimin Liu, Liang Guo, Hui Wang, Qingyan Jia, Guangle Niu, Xing Huang, Hangyue Zhou, Xiangmin Meng, Pengfei Wang, Chun-Sing Lee, Wenjun Zhang, and Xiaodong Han, *A graphene quantum dot photodynamic therapy agent with high singlet oxygen generation*, article number 4596, 2014 [63]. This work is licensed under the Creative Commons Attribution 4.0 International License.

The highest ^1^O_2_ production from illuminated GQDs was measured by Ge et al. [63]. S,N-doped GQDs showed PL emission at 680 nm, and ^1^O_2_ quantum yield of 1.3. This tremendously high photoproduction of ^1^O_2_ was explained by the multistate sensitization process. They calculated that the energy gap (ΔE_ST_) between the singlet excited state (S_1_) and the triplet excited state (T_1_) is higher than the energy needed for ^1^O_2_ formation (22.5 kcal mol^−1^). Another energy difference is large enough to provide energy for ^1^O_2_ production, and that is the transition from triplet to the ground state (ΔE_TG_). Both transitions lead to ^1^O_2_ generation. Figure 9 shows the in vivo PDT of GQDs evaluated using female BALB/nu mice with subcutaneous breast cancer xenografts as an animal model. This treatment did not allow regrowth of the tumor over 50 days.

Gamma irradiation treatment of GQDs increased photoinduced singlet oxygen production [27]. Jovanovic et al. found that gamma irradiation, in the presence of 2-propanol, produced GQDs and had a four-times greater ability to produce singlet oxygen [27]. GQDs produced from biomass were able to kill 90% of the cells after irradiation with an 808 nm laser, while the temperature rose to 49 °C [217]. GQDs generated both singlet and superoxide anion radicals when they were exposed to light [218], while a different study showed that chemical reduction improves their ability to generate ROS [219]. 

F-doped GQDs were explored as an agent in PDT, and showed a high ability of ^1^O_2_ generation under visible light (QY was 0.49) [220]. When carcinoma cells HepG2 were irradiation for 12 min and treated with 200 μg mL^−1^ of F-GQDs, 70% of the cells were dead.

To the contrary, one study claimed that single-layered GQDs with a diameter of 5 nm, and others with 20 nm, were unable to produce ROS when they were excited with a 660 nm laser or a halogen light (400–700 nm) [221]. They showed that DPBF is insoluble in water and leads to false positive results, while the photoluminescence of SOSG is quenched only by adding GQDs. Though ADMA and RNO are soluble in aqueous media, the intensity of the characteristic bands was not changed significantly with irradiation. The same study indicated that even EPR with TEMP did not improve singlet oxygen production. The authors suggested that the lack of N in the GQDs was the reason for the inability of ROS photoproduction. 

Peptide-functionalized GQDs showed an extraordinary ability to produce singlet oxygen, with a quantum yield of 0.95 [222]. With 15 min of irradiation at 450 nm, mice with malignant melanoma, injected with 4 mg kg^−1^, showed an obvious decrease in the tumor size. GQDs were decorated with europium, silver, and selenium, and also showed the ability to photogenerate singlet oxygen [223].

The papers analyzed in the present study confirm that GQDs are able to produce different ROS, and in most of the studies, singlet oxygen was the main photogenerated product, with QYs of 1.3%, 0.95%, and 0.49%. The largest ^1^O_2_ production was measured for N,S-doped GQDs, but GQDs without heteroatoms can photogenerate ROS as well.

#### Photodynamic Therapy with GQDs-Based Composites

Apart from the structural modification of GQDs, another approach to increase the efficiency of classical PDT agents, anticancer drugs, and contrast agents, is their functionalization with GQDs. These complex systems showed promising results due to more selective transport to tumor tissue, reducing the side effect of anticancer agents, and increasing the efficiency of therapy due to the high cancer cell death rate.

Nanoparticles of porous silica were encapsulated with GQDs in the complex with hypocrellin A (HA), a PDT agent, which showed phototoxic effects in cancer cells [224]. In vitro production of singlet oxygen, after only 240 s of illumination at 470 nm, was detected, while HeLa cell viability was 20%. GQDs functionalized with T_1_-weighted magnetic resonance imaging (MRI) contrast Mn_3_O_4_ and polydopamine were exposed to a 670 nm laser, inducing the death of around 50% of human lung cancer cells A549 [225]. Silver nanoparticles, functionalized with polyethylene glycol (PEG) and covered with GQDs, were employed as a drug delivery system for doxorubicin [226]. Singlet oxygen production was recorded at a light wavelength of 425 nm, and led to cell viability of 35% and 15% for HeLa and DU145 cells, respectively. 

**Figure 10:**
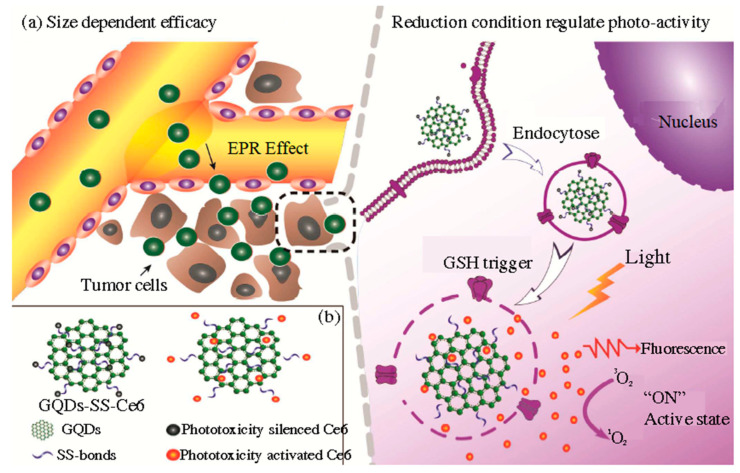
A complex of GQDs with Ce6 internalized into tumor tissue through FRET; Ce6 was released from the complex (**a**); a schematic presentation of the GQDs-SS-Ce6 complex (**b**). Reprinted with permission from *ACS Applied Materials and Interfaces 8*, by Dou Du, Kun Wang, Ya Wen, Yan Li, and Yong Y. Li, *Photodynamic Graphene Quantum Dot: Reduction Condition Regulated Photoactivity and Size Dependent Efficacy*, 3287 [228]. Copyright 2016 with permission from the American Chemical Society.

GQDs were bound, through disulfide bonds, to the classical PTD agent chlorine e6 (Ce6), and this complex was functionalized with PEG (GQDs/Ce6/PEG nanoparticles) [227]. Disulfide bonds were broken when glutathione was present, which is inside cells, and the created GQDs-Ce6 was then activated to produce singlet oxygen. When HeLa cells were incubated with GQDs/Ce6/PEG nanoparticles and illuminated at 650 nm, the cell viability was less than 20%. The material accumulated in the kidneys, and the tumor volume was reduced from 267 to 118 mm^3^. A similar glutathione-triggered complex was produced by Du et al. [228]. Due to their small size, GQDs tend to accumulate in tumor tissue through the enhanced permeation and retention effect (EPR effect), presented in Figure 10a. When the complex enters the cells, cleavage of the disulfide linker occurs due to the higher concentration of glutathione in the cell than in the extracellular space (exterior concentration is only 2 µM, while interior cytosolic concentration is 10 mM (Figure 10a). The released Ce6 recovered its phototoxicity. The complex showed higher toxicity (around 20%) than Ce6 (around 30%), and it was accumulated primarily in the tumor tissue and the liver after only 2 h, while Ce6 was detected in the other organs. 

GQD-Ce6 complexed with hyaluronic acid (HA) showed the ability to produce singlet oxygen [229]. To induce phototoxicity, irradiation with a 670 nm laser was used. Human lung cancer cells were exposed to Ce6 and the GQD-HA-Ce6 complex at a concentration of 50 μg mL^−1^, which induced the death of 50% and 82% of the cells, respectively. 

GQDs in the complex with classical PDT agents play a role in the selective delivery, and in the way they increase the anticancer efficiency, while at the same time lowering the toxic side effect of treatment. Due to the observed photoinduced toxicity of GQDs themselves, as discussed in Section 4.1, upon illumination, GQDs are able to produce ROS and probably further improve the efficiency of the therapy. 

### 4.2. Photodynamic Therapy with CQDs

Starting precursors for CDs synthesis and preparation methods affect the CDs’ properties and possible anticancer effects. Li et al. prepared CDs from tender ginger juice and showed very good potential anticancer activity in vitro, as well as in vivo, where 440 μg inhibited the growth of tumors in mice within 14 days [230]. He et al. designed new CDs with good photostability and biocompatibility, great cellular uptake, and potent cytotoxicity upon irradiation, and in vitro and in vivo experiments with a concentration of 0.2 mg mL^−1^ showed tumor growth inhibition [12]. Wu et al. developed folate-conjugated reducible polyethyleneimine passivated CDs (fc-rPEI-CDs), which could encapsulate multiple siRNAs (EGFR and cyclin B1), followed by releasing them in an intracellular reductive environment [231]. An in vitro cell culture study demonstrated that fc-rPEI-CDs are a highly biocompatible material, and a good siRNA gene delivery carrier for targeted lung cancer treatment [231]. Vasimalai et al. prepared CDs from spices (cinnamon, red chili, turmeric, and black pepper) [232]. These dots inhibited cell viability dose-dependently after a 24 h incubation period, displaying higher toxicity in LN-229 (human glioblastoma cells) than in HK-2 cells (HK-2 noncancerous cell line). The concentration of these dots was altered between 0.1 and 2 mg mL^−1^. Doping of CDs by N- and P-heteroatoms contribute to their toxicity, under 532 nm laser irradiation, against A549 cells [233].

CDs containing porphyrin are ultrasmall in size, possess good water solubility, and are photostable [234]. These dots generate singlet oxygen under irradiation and induce cell apoptosis. Thus, they inhibit the growth of hepatoma. Beack et al. synthesized conjugates from CDs, chlorine e6, and hyaluronate [235]. These conjugates were prepared by the coupling reaction of diaminohexane-modified HA (DAH-HA) with the carboxylic group of chlorine e6. Singlet oxygen generation of these conjugates is higher than that of free chlorine e6. Complete suppression of B16F10 melanoma skin cancers occurred after treatment with these conjugates irradiated by laser. The CQDs sample showed the highest photocytotoxic activity toward the Hep2c cell line (12.53 μg mL^−1^). Jin et al. reported the synthesis and anticancer cell activity of nitric oxide (NO)-releasing CQDs [236]. The anticancer activity of the NO-releasing CQDs against Pa14c, A549, and SW480 cancer cell lines proved to be dependent on both NO payloads and surface functionalizations. Primary amine-modified CQDs with NO payloads of ~1.11 μmol mg^−1^ exhibited the greatest anticancer action. The three types of CDs showed high phototoxicity for three cellular lines: human rhabdomyosarcoma (RD), a cell line derived from human cervix carcinoma Hep2c (HeLa), and a fibroblast cell line from murine tissues (L2OB) [29]. The highest photocytotoxic activity was shown in the N-doped CQDs sample, especially toward RD cells (5.35 μg mL^−1^).

Based on the observations mentioned above, we can conclude that different types of CDs, predominantly GQDs and CQDs, can be used as potent anticancer agents upon visible light irradiation. Different studies showed that the CD structure, concentration, particle size, chemical composition, and quantum yield of singlet oxygen are the main parameters that affect the potential anticancer usage of CDs.

## 5. Antibacterial PDT with GQDs and CQDs as Agent

GQDs exhibit very good photodynamic antibacterial characteristics [26,237]. However, researchers often use them to create nanocomposites with other nanoparticles, and improve their characteristics [238]. Zhang et al., for example, created a nanocomposite where GQDs acted as a shell layer covering the silver nanoparticles, which significantly improved the antibacterial properties of the nanocomposite compared to both nanoparticles alone, due to the synergistic effect [239].

N-doped GQDs showed the ability to produce both singlet oxygen and superoxide anion radical (O_2_^•−^) when they were exposed to 670 nm wavelength laser, inducing 100% *Escherichia coli* (*E. coli*) elimination, at a concentration of 1 μg mL^−1^ [240]. 

Amino-functionalized GQDs showed a superior ability to generate ROS when they were illuminated with NIR light [241]. Namely, 100% elimination of methicillin-resistant *Staphylococcus aureus* (MRSA) was achieved with 0.25 μg mL^−1^ of amino-functionalized GQDs and 800 nm light, due to ^1^O_2_ and O_2_^•−^ production. Kuo et al. also showed that GQDs with amino functional groups can be used as an antibacterial and a contrast agent [242]. Again, both ^1^O_2_ and O_2_^•−^ are produced during two-photon excitations, with the light at 800 nm. *Bacillus subtilis* (*B. Subtilis*) was treated with 6 μg mL^−1^ of amino-N-doped dots functionalized with antibodies, and the application of light resulted in 100% bacterial cell elimination. 

GQDs were modified by establishing π–π interactions with phthalocyanine derivates, and showed the ability to produce singlet oxygen [243]. These complex nanomaterials showed antibacterial activity toward Staphylococcus aureus at a concentration of 10 μM; the reduction was 9.68 log, with 0% cell survival, when they were exposed to 670 nm wavelength light. The singlet oxygen quantum yield was 0.79 for the most efficient compound. 

Composites based on GQDs and AgNPs seem to be very efficient materials for the treatment of *E. coli* and *S. aureus* due to the ability of this composite to produce ROS [244]. This material caused the death of more than 80% of *E. coli* cells, at a concentration of 1 μg mL^−1^. When the bacterial cells, treated with GQD-AgNPs, were exposed to light (808 nm, 2 W cm^−2^), the temperature increased from 2 to 22 °C, when concentrations were 90 to 2700 μg mL^−1^. It was observed that after only 10 min of NIR illumination, at a concentration of 2 μg mL^−1^, the viability of bacteria was 0%. Herein, the mechanism of bacterial cell damage was explained by ROS production and damage to the bacterial membrane.

The combining of GQDs and AgNPs as a PDT agent resulted in the production of a photothermal agent. Synergistic effects of both materials lead to a temperature increase up to 40 °C, as well as ROS production during irradiation, and rupturing of bacterial cell membranes [245].

A therapy for periodontitis using GQDs as a PDT agent was recently explored [246]. GQDs functionalized with curcumin were able to produce ^1^O_2_ under illumination with a blue LED lamp at 435 nm, and an intensity of 1000–1400 mW cm^−2^. Bacterial biofilms that cause periodontitis are produced by *A. actinomycetemcomitans, P. gingivalis,* and *P. intermedia.* Showing antibiofilm activity, ROS production was detected from GQDs loaded with curcumin at 405 nm [247]. Bactericidal effects against Gram-positive bacteria (*S. aureus* and MRSA), two Gram-negative bacteria, *Pseudomonas aeruginosa* (*P. aeruginosa*) and *E. coli*, and the yeast *C. albicans,* were reported. 

GQDs and hollow mesoporous silica nanoparticles were shown to be efficient agents in PDT of a wound [248]. Both *E. coli* and *S. aureus* were treated with the composite, in concentrations of 4 to 12 μg mL^−1^ and light (LED lamp, 5 W at a distance of 40 cm) for 15 min. The wound area was 100% closed after composite and light treatments, while only 55% were closed in the control group. 

**Figure 11:**
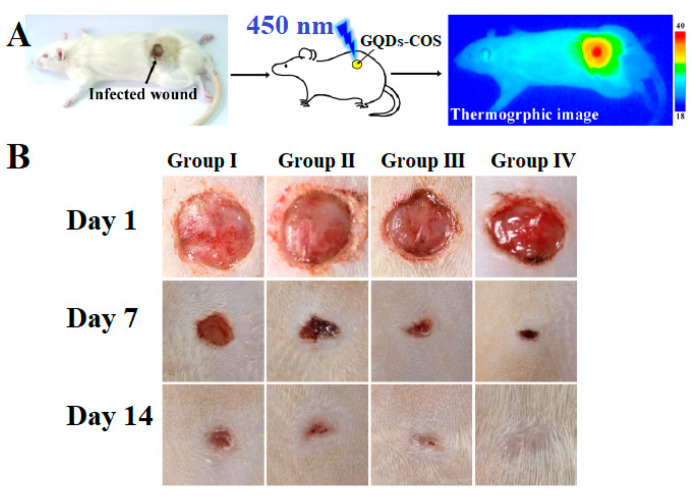
Image of wounds and thermographic image of wounds after treatment with GQD-Chi composite and illumination (**A**). In part (**B**), photographs of the wound at different stages are presented. Reprinted with permission from *ACS Applied Materials and Interfaces*, 12, Yanmei Shi, Cui Cheng, Zongkai Shi, Mingli Jiao, Fengyi Cao, Zhenlong Xu, Xiumin Li, and Junxia Zhang, *Augmented Graphene Quantum Dot-Light Irradiation Therapy for Bacteria-Infected Wounds,* 40153 [249]. Copyright 2020 American Chemical Society.

GQDs functionalized with chitosan (Chi) showed PDT, photothermal, and antibacterial effects [249]. The bacteria-infected wound was treated with GQDs-Chi and light at 450 nm. The synergistic effect resulted in high antibacterial efficiency, due to photoinduced ROS production from GQDs, and the antibacterial effects of chitosan. The positively charged chitosan chain attracted bacterial cells through electrostatic interactions. Both *E. coli* and *S. aureus* were inhibited when they were treated with 100 μg mL^−1^, and the effect was two times higher when samples were irradiated. An in vivo study showed that a rat wound, infected by *S. aureus,* healed completely after treatment with a GQDs-Chi composite and light (Figure 11). Functionalized GQDs, in combination with blue light, decreased the time for wound closing (Figure 11A), causing both photoinduced ROS production and a photothermal effect. After 7 days, these effects led to the almost complete closing of the wounds, and no signs of infection (Figure 11B). 

The mechanism of the antibacterial action of CDs is their specific interaction with the cell wall of bacteria; however, the generation of ROS, when exposed to light of a certain wavelength, is more dominant. Firstly, the adhesion of the CQDs to the bacterial surface occurs, then, upon light irradiation, the CQDs start producing ROS, which leads to oxidative stress, damage to the DNA/RNA, proteins, and other biomolecules of bacterial cells [20,250]. Apart from the oxidative stress induced by the formation of ROS, several other antibacterial mechanisms of CQDs were noted, such as DNA binding, membrane destabilization, physical and mechanical damage, inhibition of bacterial metabolism, etc.; therefore, it is complicated for bacteria to develop resistance [250]. These mechanisms depend on the concentration and size of CDs, their functional groups, and surface charge, but also the cell wall properties of the bacteria.

Lipopolysaccharides and lipoteichoic acids on bacterial membranes are negatively charged, thus, CQDs modified with a positive charge leads to electrostatic interaction and antibacterial activity [251,252,253]. Shahshahanipour et al. developed high fluorescence CDs that kill Gram-positive and Gram-negative bacteria in much lower concentrations than antibiotic drugs [254]. N-doped CQDs, synthesized by a one-step chemical route in 0.5 mg mL^−1^ concentration, affect the cell structure of *Staphylococcus aureus* (*S. aureus*) and MRSA, but do not combat *E. coli* [255]. Three types of photoinduced CDs (GQDs, CQDs, and N-doped CQDs) showed high antibacterial activity against bacteria-caused nosocomial infections: *Enterobacter aerogenes* (*E. aerogenes*), *Proteus mirabilis* (*P. mirabilis*), *Staphylococcus saprophyticus* (*S. saprophyticus*), *Listeria monocytogenes* (*L. monocytogenes*), *Salmonella typhimurium* (*S. typhimurium*) and *Klebsiella pneumonie* (*K. pneumonie)* [28]. Park et al. prepared CDs by plasma treatment using polyethylene glycol as a precursor, which yielded a very potent antibacterial agent against *E. coli* and *Acinetobacter baumannii* (*A. baumannii*) [256]. Bing et al. [10] investigated the antibacterial activity of CDs with different surface charges [253]. According to their findings, uncharged CQDs had no antibacterial effect, while positively and negatively charged CQDs had a bactericidal effect on *E. coli*. After CQDs-induced bacterial cell death, DNA breakage, chromosomal condensation, and loss of structural integrity occurred, indicating that *E. coli* had the biochemical mechanism to promote their termination, once cell death had been triggered by CQDs.

Surface functionalization impacts the capability of CQDs to interact with other organic molecules, drugs, and procaryotic and eucaryotic cells. Therefore, it significantly affects their antibacterial activity, cytotoxicity, and determination of cellular uptake. The antibacterial efficacy of S-doped CQDs and N-doped CQDs were compared by Travlou et al. [95]. Their findings demonstrated that N-CQDs had a significantly higher antibacterial efficacy, which was related to both the production of ROS and their positively charged amine and amide groups. S-doped CQDs, however, showed a meaningfully lower antibacterial effect. Due to the dissociation of sulfonic/carboxylic groups and sulfates, they were primarily negatively charged, and showed size-dependent rather than surface charge-dependent suppression of Gram-positive bacterial growth. Antimicrobial activity of CQDs against Gram-positive (*S. aureus* and *L. monocytogenes*) and Gram-negative pathogens (*E. coli* O157:H7 and *S. typhimurium*) was improved by N and/or S doping, which increased as the ratio of S decreased [257]. By adding N-containing 2,2′-(ethylenedioxy)bis(ethylamine) (EDA) or polyethyleneimine to carbon nanopowder, a carbon source, CQDs with excellent antibacterial potentials were prepared [257]. Chai et al. showed that P-doped CQDs prepared by simple hydrothermal treatment of m-aminophenol and phosphoric acid had effective antibacterial activity against *E. coli*) and *S. aureus* [258]. The minimal inhibitory concentrations (MICs) of P-doped CQDs were 1.23 mg mL^−1^ for *E. coli* and 1.44 mg mL^−1^ for *S. aureus*. 

Photodynamic antibacterial properties directly impact ROS production by CQDs doped with F and Cl, compared with undoped nanoparticles [189]. 

Li et al. examined the antibacterial activity of fluorescent spermidine-capped CQDs (Spd-CQDs) against *MRSA*, *E. coli*, *S. aureus*, *B. subtilis*, and *Pseudomonas aeruginosa* (*P. aeruginosa*) pathogens. They demonstrated Spd-CQDs’ highly effective antibacterial properties and great biocompatibility [252]. The authors concluded that Spd-CQDs induced significant damage to the bacterial membrane. Nitrogen-doped carbon quantum dots (NCQDs), synthesized by a one-step chemical route, in 0.5 mg mL^−1^ concentration, affect the cell structure of *S. aureus* and MRSA, but do not combat *E. coli* [255]. The authors suggest that the reason for this result is the difference in the cell wall between the Gram-positive *S. aureus* and Gram-negative *E. coli* strains. The cell wall of Gram-positive bacteria is porous, due to the thick layer of peptidoglycan in the plasma membrane; thus, it promotes the interaction between the NCQDs and *S. aureus* or *MRSA*. The cell wall of Gram-negative bacteria consists of an outer membrane and an intermittent peptidoglycan layer, preventing the adhesion of NCQDs to their cell wall [255].

CDs can be encapsulated into various polymer films, and form very potent photodynamic antibacterial surfaces [112,113,259]. These composites showed a high level of antibacterial activity under blue light irradiation against *S. aureus*, *E. coli*, and *K. pneumonie*. Gamma ray irradiation of these composites induced changes in morphology and chemical composition, and contributed to enhanced antibacterial activity toward *S. aureus* and *E. coli* [114]. CQDs thin films showed very good antibacterial activity against *S. aureus* and *E. coli* [109]. *E. coli* is more sensitive to the surface of CQDs thin films, compared to *S. aureus*. The obtained results indicated that a large number of *E. coli* bacteria strains are dead after 1 h. GQDs incorporated into bacterial cellulose hydrogels have good biocompatibility, and show significant inhibition of *S. aureus* and *Streptococcus agalactiae,* and bactericidal effects against *MRSA*, *E. coli*, and *P. aeruginosa* [260].

The effect of the particle size on the antibacterial activity of CQDs was investigated by Sun et al. [261]. Small ~2, medium ~3.9, and large particle size ~5.3 nm were studied, and antibacterial activity was increased with the decrease of the particle size. The authors confirmed the insertion of CDs into the cell wall of the bacteria, and then, the cellular uptake and distribution were different for each group of CDs. 

Table 5 summarizes the antibacterial activity of different types of CDs.

**Figure 12:**
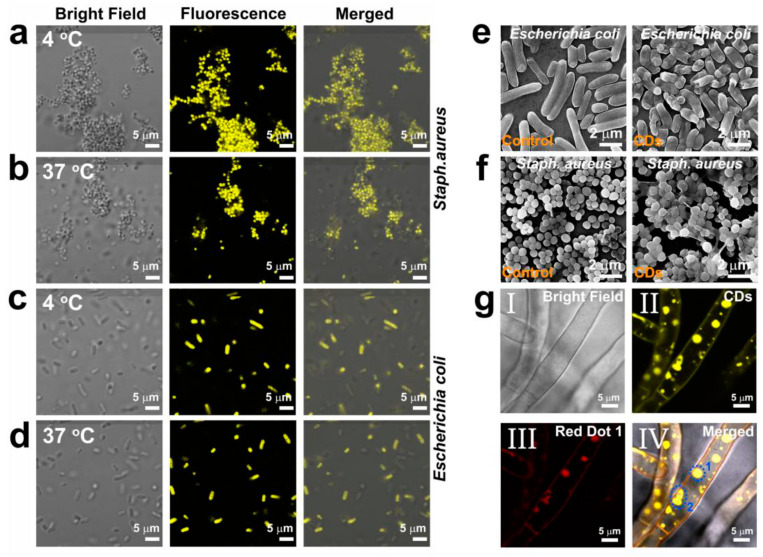
(**a**,**b**) Confocal laser scanning microscopy (CLSM) images of *S. aureus* (Gram-positive) treated with CDs (25 µg mL^−1^) for 1 h at 4 and 37 °C. (**c**,**d**) The CLSM images of *E. coli* (Gram-negative) treated with CDs (25 µg mL^−1^) for 1 h at 4 and 37 °C. (λ_ex_ = 405 nm; emission was collected at 415–550 nm, for both strains). (**e**,**f**) SEM images of *S. aureus* and *E. coli* after incubation without and with CDs at 60 µg mL^−1^ for 12 h, respectively. (**g**) CLSM images of *Rhizoctonia solani* treated with CDs and Red Dot 1 for 30 min at 37 °C: (I) bright field, (II) CDs (200 µg mL^−1^; λ_ex_ = 405 nm; emission was collected at 415−550 nm), (III) Red Dot 1 (200× in water; λ_ex_ = 543 nm; emission was collected at 580–750 nm), and (IV) merge of images (the blue traces marked 1 and 2 are nucleus and ruptured nucleus, respectively). Reprinted with permission from *ACS Applied Materials and Interfaces*, by Hao Li, Jian Huang, Yuxiang Song, et al., *Degradable Carbon Dots with Broad-Spectrum Antibacterial Activity*, 10, 26936 [262]. Copyright 2018 American Chemical Society.

To better understand the antibacterial and antifungal action of CDs, researchers investigated the cellular uptake of CDs through fluorescence microscopy. In the recent work by Li et al. [262], the fluorescence of CDs within bacteria was visualized at the excitation wavelength of 405 nm (Figure 12a–d). They also confirmed that the CDs can cover the external surface of bacteria cells, making the surface rougher, which is illustrated in SEM images (Figure 12e,f). This further leads to indirect toxicity, caused by isolating the bacterial cells from the growth medium. Additionally, the authors used a confocal microscope to illustrate how CDs inhibited the growth of the fungus *Rhizoctonia solani,* by entering directly into the nucleus and disrupting it (Figure 12g).

**Table 5:** CDs as antibacterial agents.

Material	Diameter	Toxicity	Mechanism	Observations
+ charged, −charged, and 0 CDs [253]	2.69–3.04	+ CDs: 100%, − CDs: ~80% 0 CDs: ~15% viability loss of *E. coli* incubated with 300 µg mL^−1^ for 6 h.	ROS production disrupting cytoplasmic membrane by + charged CDs	+ CDs had the highest antibacterial activity, while 0 charged had the lowest
CQD-EDA [263]	5 nm	*E. coli:* ~95% viability loss in the light conditions after 6 h	ROS production under visible light	The first report on the visible/natural light-activated antibacterial activity of CDs
GQDs [26]	20–67 nm	*E. coli:* 80% MRSA: 90% viability loss;	ROS production under blue light (470 nm)	Fast antibacterial action, only 15 min of exposure
N-GQD [240]	8 nm, height ~ 1.03 nm	Killing 100% of *E. coli* in only 3 min of exposure	ROS production under 670 nm laser irradiation, the synergistic effect of ROS and RNS (reactive nitrogen species)	Higher nitrogen content in GQDs leads to more efficient PDT
GQD, CQDCA, and CQDNH [28]	GQD: 14 nm; CQDCA: 22.5 nm; CQDNH: 12.5 nm	*E. coli*, *E. aerogenes*, *P. aeruginosa*, *K. pneumoniae*, *B. subtilis* MIC: 3.905–250 µg mL^−1^	ROS production under blue light (470 nm)	N-CQDs showed the best antibacterial properties
CDs three groups according to sizes [261]	Small (s-CGCD): ~2 nm Middle (m-CGCD): ~3.9 nm Large (l-CGCD): ~5.3 nm	For *E. coli*: the concentration of s-CGCD ˃ 100 µg mL^−1^, for m-CGC and l-CGCD ˃150 µg mL^−1^ For *S. aureus*: 50, 75, and 100 µg mL^−1^ for s-CGCD, m-CGCD, and l-CGCD	No ROS production. The mechanism includes destroying the cytoplasmic membrane of bacteria by causing the leaking of cellular components	The antibacterial effect was increased with the decrease in particle size
Curcumin carbon dots from curcumin, neutral red, and citrate (Cur-NRCQDs) [264]	~3.83 nm	Cur-NRCQDs inactivated 100% *S. aureus* and *E. coli* at concentrations of 10 and 15 μM	ROS production under the xenon lamp 555–850 nm	Cur-NRCQDs efficient against biofilms
Graphitic carbon nitride quantum dots: g-CNQDs [265]	2–7 nm	Inhibition of ~99% of *E. coli* and ~90% of *S. aureus* at a concentration of 100 µg mL^−1^	ROS production under visible light	Antibacterial activity of g-CNQDs was equivalent to silver nanoparticles
CDs from vitamin C [262]	~5 nm	Killing 100% of a broad spectrum of bacteria at a concentration of 100 µg mL^−1^ at 150 µg mL^−1^, inhibiting the growth of fungus	CDs can enter the bacteria by diffusion, destroy the cell wall, bind to the DNA and RNA of bacteria, and finally kill them	These CDs could be degraded into CO_2_, CO, and H_2_O under visible light in the air after 20 days

## 6. Cytotoxicity of GQDs and CQDs

The biosafety of CDs is very important when their biomedical application is considered. The toxicological profile of CDs, or side effects on host cells, is one of the first biological analyses for potential use in medicine [266]. The interaction mechanism involves receptor-mediated endocytosis or direct binding to membranes, and in some cases might provide obstruction of channel proteins, which leads to blocking the entry of important metabolites. The contact area of CDs is in a positive correlation with biocompatibility. The toxicity evaluation depends on surface charges, concentration ranges, and safe doses [267]. 

### 6.1. Toxicity of GQDs

Numerous studies were conducted investigating the toxicity of GQDs [268,269,270]. The summarized results are presented in Table 6. 

It was observed that GQDs are internalized into cells faster, and show lower cytotoxicity compared to graphene oxide (GO), with sizes from several hundreds of nm to several microns [269]. Pioneering studies in this field showed that GQDs internalize into human gastric cancer MGC-803 cells and accumulate in the cell cytoplasm, inside the endoplasmic reticulum (ER) [269]. It was suggested that the mechanism of internalization is caveolae-mediated endocytosis, as one of the possible ways for molecules to access the Golgi and the ER. Cellular uptake of GQDs has been studied in three different cell lines: macrophages, endothelial cells, and models of cancer cells [271]. This study confirmed the caveolae-mediated endocytosis pathway, followed by clathrin-mediated endocytosis. The authors also showed that GQDs have a small effect on cell proliferation, internal cellular ROS level, and mitochondrial membrane potential. 

The second study encouraged further investigation of GQDs application in different fields, due to providing proof of biocompatibility of GQDs in an in vivo study [270]. Namely, carboxylated GQDs accumulated in tumor tissue and other organs, such as the liver, spleen, and kidneys, after 24 h of application, and no toxic effects were observed at doses of 5 and 10 mg kg^−1^. Another study showed fast elimination of GQDs through the kidneys, while no signs of toxicity or inflammation were detected, even after multiple administrations [268]. The higher accumulation of GQDs functionalized with PEG is due to the enhanced permeability and retention effect, characteristic of tumor tissues, while in the kidneys the highest concentration was detected. Even at doses of 20 mg kg^−1^ administrated every second day for two weeks, there were no toxic effects. Qin et al. showed that GQDs affect the immune system and, at low concentrations, cause the higher expression of tumor necrosis factor-α (TNF-α), interleukin-1β (IL-1β), and IL-8, while when the concentration of dots is higher, they induced cytokine production [272]. The authors observed that GQDs induce the production of ROS inside cells, and immunotoxicity in human macrophages, through p38 MAPK and NF-kB signaling pathways. The authors observed an inflammatory response, apoptosis, and autophagy at a concentration above 200 μg mL^−1^. Chanrda et al. showed that intracellular production of ROS was reduced when GQDs were functionalized with PEG, revealing this approach as a possible strategy to further increase GQDs’ biocompatibility and safety [273]. 

**Table 6:** Toxicity of GQDs with different sizes and structures.

Sample	Structure	Size	Toxicity
GQDs [269]	Oxygen-containing, no specific data	20 nm	MGC-803 and MCF-7, 80% cell viability at 400 μg mL^−1^
GQDs [270]	Carboxylated GQDs	5 nm	KB, MDA-MB231, A549, and MDCK 80% cell viability at 500 μg mL^−1^
GQDs [268]	GQDs-PEG, 36% O	5 nm	20 mg kg^−1^ every second day for 14 days
GQDs [272]	O-GQDs, C–O, C=O	1.5–4 nm	Slight toxicity on macrophage at 400 μg mL^−1^
GQDs [273]	PEG-GQDs	6.6 nm to 88 nm PEG	Not toxic on HeLa cells at 8 μg mL^−1^
N-GQDs [40]	NH_2_ groups, pyrrolic, pyridinic	3.5 nm	No toxic effect on HeLa cells at 100 μg mL^−1^ (72 h)
GQDs [267]	NH_2_, COOH, and CO–N (CH_3_)_2_		Low cytotoxicity to A549 at up to 200 μg mL^−1^
N-GQDs [274]	NH_2_ groups, pyridinic	2.3–6.4 nm	No toxic effects on HeLa up to 200 μg mL^−1^, low effect on zebrafish embryos and larvae
Chiral [275]	L- or D-cysteine moieties attached to GQDs	3–7 nm	HepG2 cells to l/d-GQDs general biocompatibility and d-GQDs accumulate in cellular membrane
N-GQDs [276]	N-doped	5.1 nm	No hemolysis and release of ATP in RBCs, up to 200 μg mL^−1^
N-doped GQDs [277]	N-doped, NH_2_ groups	2.1 nm	No toxic effect SW480 cells at 0−1000 μg mL^−1^
P,N-doped [278]	41.79% C1s, 43.65% O1s, 5.85% N1s, 8.71% P2p	4.2	90% T24 cell viability at 20 to 100 μg mL^−1^
GQDs [279]	C 51 %, O 42%, N 8%	20	No toxic effect at 1000 μg mL^−1^
N-doped [280]	Amino, and pyrrolic groups	2.3–5.0	100% HeLa cells viability at 200 μg mL^−1^ after 48 h
GQDs [271]	C, O	4–6	80% cell viability at 1 mg mL^−1^
FA-GQDs [281]	Folic acid encapsulated N-GQDs	33.59	80% HeLa cells viability at 2.0 mg mL^−1^
GQDs [282]	/	/	In vivo, mice, 300 mg kg^−1^

All of these studies indicated that GQDs are able to enter different types of cells. They did not show toxic effects, even at very high doses, in live organisms such as mice or zebrafish, and they did not show genotoxic effects in the first, second, and subsequent generations. These studies suggest that GQDs are nontoxic and biosafe materials. 

### 6.2. Toxicity of CQDs

Bagheri et al. confirmed positive dose-dependent toxicity of CQDs on yeast cells [283]. 

CDs prepared with Trapa Bispinosa peel extract showed more than 80% MDCK cell survival at concentrations of 1–4 μg mL^−1^ [284]. In the study of Lou et al., CDs from aqueous extracts of Radix Puerariae Carbonisata, at a very high concentration (1000 mg mL^−1^), showed 80% viability on RAW 264.7 murine macrophage cells from blood [285]. CDs obtained from ginger juice showed more than 60% viability of human cervical cancer cell lines (HeLa), human lung cancer cell lines (A549), human breast cancer cell lines (MDA-MB-231), and HepG2 cells [230]. 

In a context of CDs as photosensitizers, one of the main drawbacks is ROS production, which may disrupt cell signaling and DNA molecules, causing toxicity. The surface area is in a positive correlation with ROS production. Additionally, chemical structure, size, solubility, pH, light wavelength, and surface functional groups have a strong impact on ROS levels [286]. 

For use in photodynamic therapy, GQDs have high potential due to their low toxicity and photostability [63,287]. Through the process of doping and codoping with nitrogen (N), boron (B), phosphorus (P), and sulfur (S), or their combination, the toxicity levels decrease [163,288,289,290,291]. CQDs thin films, deposited by the Langmuir–Blodgett method, showed low-level cytotoxicity on mouse embryonic fibroblast cell lines [109]. Cytotoxicity of these films changed slightly during 6 h of blue light irradiation. Cell viability tests on NIH/3T3 and A549 cell lines showed that CQDs/polyurethane nanocomposites were not toxic toward NIH/3T3 cells, regardless of the extract concentration [259]. The same nanocomposites showed mild and moderate cytotoxicity toward A549 cells, only when the extract concentrations were 75 and 100%. Gamma-irradiated CQDs/polyurethane nanocomposite did not show any cytotoxicity toward Hela cells [114]. However, for U-87 MG cells, only nanocomposites irradiated at a dose of 200 kGy exhibited mild or moderate toxicity, and only when extract concentrations were 75 and 100%, respectively. It should be noted that U-87 MG cells are generally more sensitive than HeLa cells.

### 6.3. In Vivo Toxicity and Genotoxicity of GQDs and CQDs

The toxicity and genotoxicity of GQDs were studied on zebrafish, as one of the most promising in vivo models [274,292,293]. One study showed that GQDs may induce significant transcriptomic responses in zebrafish larvae [294]. Activator protein 1 (AP-1) is connected to apoptosis and could be activated by GQDs, indicating possible risks of bleeding. The effect of GQDs on the health of male mice, and their first and second offspring, was also evaluated [282]. Both oral and intravenous injections do not affect the function of the reproductive system organs or testosterone levels in male mice; the first, second, and subsequent litters from female mice treated with GQDs were healthy, which indicated that this nanomaterial is not toxic to germ cells, and proves the fast elimination after administration via the urinary and gastrointestinal tract. Male mice were treated at very large doses (150 mg kg^−1^ intravenous and 300 mg kg^−1^ oral doses for 30 days), and the function and physiology of the liver and kidneys, as well as the hematology parameters, were normal. This study proved the biosafety of GQDs in mice and their offspring. 

**Figure 13:**
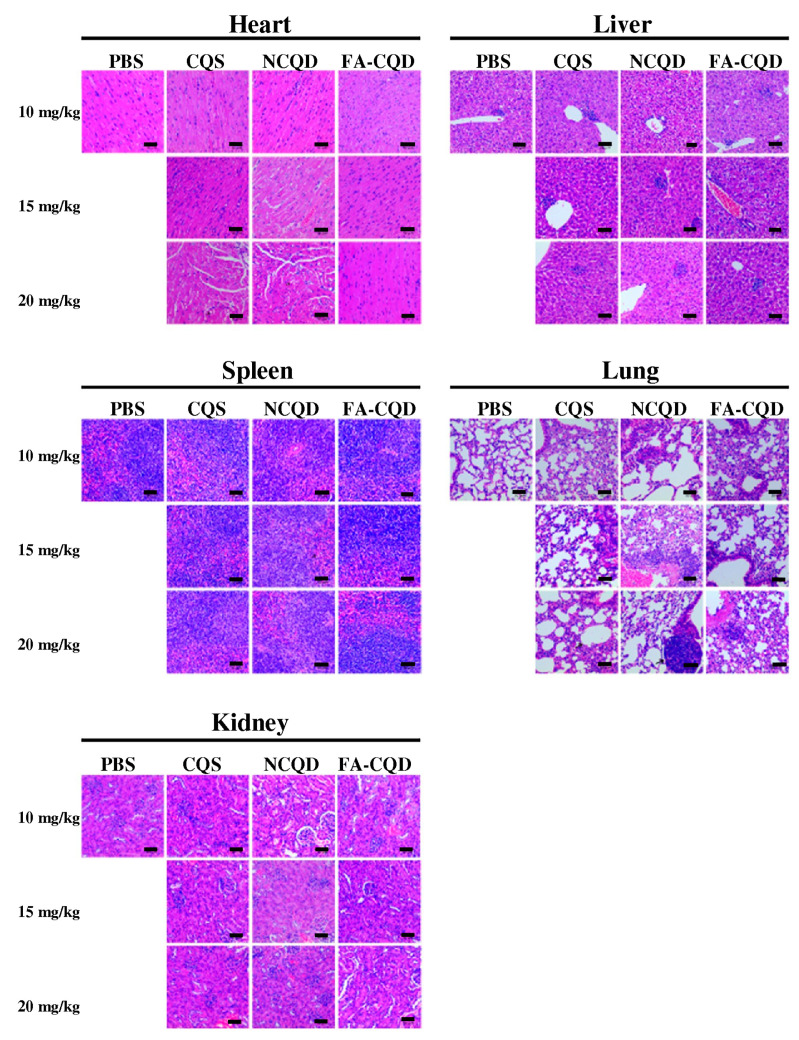
Histological analysis of the CQD materials to evaluate toxicity. Histological study of the heart, liver, spleen, lung, and kidney of mice at 14 days after intravenous injection of the CQD materials at different concentrations (scale bar = 50 mm). Reprinted from *Chinese Chemical Letters*, 31, by Shu Zhang, Xibo Pei, Yiyuan Xue, Jingyuan Xiong, Jian Wang, *Bio-safety assessment of carbon quantum dots, N-doped and folic acid modified carbon quantum dots: A systemic comparison*, 1654, copyright 2020, with permission from Elsevier [296].

In preclinical investigation, it is essential that in vivo studies of CQD materials do not cause side effects, and also do not show toxicity. In vivo studies with histological analyses, as well as complete blood panels and time course blood chemical analysis, revealed that these materials are safe when animals are treated with a dose of 20 mg kg^−1^ for 3 months [295]. The study examined, in vivo, the dosage up to 20 mg kg^−1^ in mice; compared to the control group, the lower doses did not show any obvious pathological change or malformation in organs, as shown in Figure 13 [296].

## 7. Current Challenges and Future Prospects

The future of CDs in photodynamic therapy is certainly bright and wide. Apart from developing and improving these materials as new, efficient PDT agents, new fields are developing as well. Thus, the development of new types of plastic composites that are able to sterilize themselves is a fast-growing field, with potential applications in medical equipment, as well as everyday life.

Before these nanoparticles find their place in the market, hospitals, and our homes, several issues must be resolved first.

The first issue is related to the difference in properties between dots that are produced by different synthetic routes. In this review, we noticed that GQDs that had the same size and similar structure show different optical properties. For instance, in Table 2, N-doped GQDs, with very similar diameters, around 4 nm, and pyrrolic, pyridinic, and amino nitrogen in their structure, showed emission in infrared, between 624 and 532 nm, and at 465 nm [61,68,74]. The origin of the photoluminescence of carbon-based nanoparticles is still unclear, and further investigation of the structure must be conducted. The lack of missing puzzles is confusing scientists and leading to a situation where each method leads to the production of different dots, although they appear structurally and morphologically similar. New instrumental methods must be applied in order to resolve the structure behind those particles.

The second issue is related to the price, production yields, and time-consuming purification. Carbon nanoparticles were usually produced in low synthetic yield, and most often the dots were isolated in dialysis, which takes a lot of time. To be transferred to industry, production must be far more efficient.

Another very important issue is the reliability of methods for the detection of ROS production. In this review, we indicated which method was used to investigate ROS generation. One recent study reported that reliability is connected with the optical properties of nanoparticles, and could lead to false results [221]. Although there are procedures developed for the detection of different radicals produced during illumination, these procedures were originally established for the analysis of molecules of photosensitizers. Nanoparticles possess many different properties; for instance, they could not be dissolved but they are instead dispersing. This might lead to changes in absorbance and luminescence. 

On the other side, studies of the toxicity of carbon-based nanoparticles generally agree that these nanoparticles are nontoxic and biosafe. In vitro studies of cytotoxicity showed that GQDs are mainly nontoxic toward different cell lines. At very high concentrations, such as 750–1000 μg mL^−1^ for N-doped GQDs [279], nondoped GQDs at 1000 μg mL^−1^ [271], or folic acid-functionalized GQDs at concentrations up to 1000 μg mL^−1^ [281], cell viability was 80% or higher. It was reported that dots can induce cytokine production and ROS production inside cells [272], while another study proved that passivation with PEG improved GQD biocompatibility, and reduced ROS production [273]. The majority of studies showed that GQDs are not entering the cell nucleus, which is related to potential genotoxicity. Further studies must be conducted. Similarly, in vitro studies proved CQDs to be nontoxic, or to have low toxicity, toward different cell lines, depending on their concentrations. In a recent study, CDs at very high concentrations (1000 mg mL^−1^), showed 80% viability on RAW 264.7 cells [285]. While in another study, a significantly lower concentration of CQDs (1.11 mg mL^−1^) exhibited a very high suppression of growth of human hepatocellular carcinoma (HepG2) cells, while at the same time, they exhibited low toxicity towards mammary epithelial cells (MCF-10A) and mouse liver cells (FL83B) [230]. The in vivo studies in mice confirmed their safety, at the dosage of 20 mg kg^−1^ for 3 months [295,296]. 

**Figure 14:**
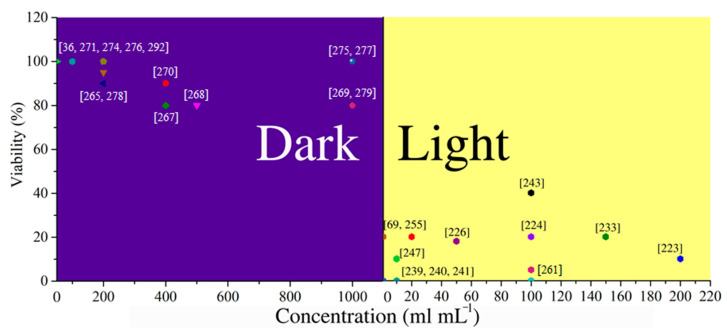
Cytotoxicity of carbon-based nanoparticles in the dark (violet, left panel), and under light exposure (yellow, right panel). Dots in the graph indicate the values of cytotoxicity as a function of studied doses of the C/GQDs, including the appropriate references [37,69,223,224,226,233,239,240,241,243,247,255,261,265,267,268,269,270,271,274,275,276,277,278,279,292].

The toxicity of carbon-based quantum dots was summarized in Figure 14. It is clear that the majority of studies proved that they are nontoxic without light, but when different wavelengths of light were applied, they become toxic. 

One of the most intriguing features is their ability to produce ROS when they are illuminated. These safe and nontoxic materials become extremely toxic and fatal when they are exposed to light. The toxic effects were documented in cancer, as well as bacterial cells. Thanks to these remarkable properties combined with chemical stability, tunable solubility, and others, these new materials are opening doors for new approaches to:

- The development of anticancer and antibacterial medication;
- Establishing new procedures for treating these conditions;
- Creating the foundation for new products, such as antibacterial plastics, for medical and domestic usage.

Carbon-based dots show advantages compared to photofrin and hematoporphyrin, which show dark cytotoxicity, cutaneous phototoxicity, and low solubility [297]. Although second-generation PSs (5-aminolevulinic acid, benzoporphyrin, chlorin, and phthalocyanine) are more soluble, and less toxic in the dark, low stability was an issue. [298,299]. Third-generation PSs are under development, combining liposome-, micelle-, quantum dot-, dendrimer-, magnetic gold-, and carbon-based nanoparticles [300]. Although this approach should lead to an efficient and safe PDT agent, the increasing number of synthetic steps, and the price of the resulting agent, is becoming a limiting issue. 

The development of carbon-based, as well as other types of nanoparticles, leads to the construction of new multifunction nanosystems, that simultaneously act as bioimaging and therapeutic agents [301].

In the war against pathogens, new products, such as lith-triggered antibacterial coatings, are rapidly developing using various carbon-based and other nanomaterials, as well as copolymers [114,302]. Are we entering the era of carbon-nanoparticle products? It is unquestionably highly probable! Positive ecological aspects of their synthesis, such as the possibility to produce dots from plants, biowaste, and biomolecules, and their low price, may be the driving force toward the development of a new branch of the smart material industry, with carbon nanoparticles as a key component. 

Will the future be brighter with carbon dots? If they are illuminated, it most certainly will be. 

## Figures and Tables

**Table 1:** Typical groups of CDs.

Types	GQDs	CQDs	CNDs	CPDs
Structure	Graphene sheets; disc-like shape; chemical groups on the edge or within the interlayer defect	Spherical and possess obvious crystal lattices and chemical groups on the surface	High carbonization degree with some chemical groups on the surface	Polymer/carbon hybrid structure comprising abundant functional groups/polymer chains on the surface and a carbon core
Precursor	Graphite, carbon nanotube	Organic molecules	Organic molecules	Organic molecules, polymers
Crystallinity	yes	yes	no	no
Polymer content	no	no	no	yes
Synthesis	Top-down	Bottom-up	Bottom-up	Bottom-up
Properties	Quantum confinement effect and edge effect	Intrinsic state luminescence and quantum confinement effect	Photoluminescence mainly originates from the defect/surface state and subdomain state within the graphitic carbon core, without the quantum confinement effect of the particle size	High oxygen/nitrogen content, excellent solubility in polar and nonpolar solvents, outstanding photoluminescence, and singlet oxygen quantum yields

**Table 2:** GQDs properties: position of emission center (Em in nm) and colors, PL QY, diameter and height, and main structural characteristics.

GQDs	Em (nm)	QY (%)	Diameter/Height	Structures (Identified Groups in at. % Where It Is Available)
N-GQDs [61]	Near-infrared	35		pyrrolic, pyridinic N, –NH_2_
N-GQDs [62]	698	62	2.7/0.7 nm	10.8% N, aromatic, –NH_2_
S,N-GQDs [63]	680	5.4 ^1^	2–6 nm	1.6% N, 5.8 S, 68.2 C, 24.4 O, C–O, C–S, C–N
N-GQDs [59]	528	-	2.45 nm	–NH_2_
S,N-GQDs [64]	610	-	3 nm	
S,N-GQDs [65]	800–850	54.5	4.7 nm	60 C, 5.4 N, 34.6 O
B-GQDs [66]	617 541	17.1 99.8	4 nm	
GQDs-PEG [67]	610	6	2.75 nm	GQD–C(O)NH–PEG
N-GQDs [68]	532 to 624	29–35	3.9 ± 0.6 nm	pyridinic, graphitic, pyrrolic N
F-GQDs [69]	455 551	56.7	6.1 nm, 2 to 4 layers	C–F and C–F_2_ bonds
N-doped (red) S,N-doped green S,N-doped blue [70]	600 540 430	24.2/red 19.7/green 20.2/blue	4.1 nm/r 3.0 nm/g 3.1 nm/b 0.8–0.9 nm height	
S-doped [71]	460			
GQDs [72]	520–620	1.1 0.89 0.65 0.38	4.5 ± 1.2 16 ± 3.3 41 ± 6.4 70 ± 15 nm	C and O
GQDs GQD- PEI 1800 GQD-PEG600 [73]	550 445 622	-	2.4 6.0 57.3 nm	Csp^2^, –COOH, –OH, –NH_2_, –CONH–
GQDs N-doped B-doped [74]	455 465 535	-	4.3 nm	C=C bonds, C=O, C–O, C–N, pyridinic and pyrrolic N, BC_3_, BC_2_O, BCO_3_
N-doped [75]	520	13.8	2.3 nm	Csp^2^, COOH, COC, C–NH_2_, CO–NH–
N-doped [76]	451	41.8	3 nm	C=C, C–N, C–O, C–NH_2_, O=N–C groups
N-doped [77]	447	54	2.65 nm	60.01% C, 36.42 O, 3.57 N
S,N-doped [78]	448	-	3.13 nm	81.1% C, 9.9 N, 4.3 S, 4.7 O, f O–H, N–H, C=O, C–O–C, N=C=S, C–S
GOQDs [79]	400–600	-	10 nm	5.6% C=C, 16.5 C=O, 6.1 C–OOH, 71.8 C–OH
S-doped [80]	450–530	11	3 nm	C–C, C=C, C–S, C=O, –S^2−^ oxidized S species (–SO_n_^−^)

^1^ Measured using integration sphere.

**Table 4:** Values of singlet oxygen quantum yield for selected CDs.

Title	ΦΔ (%)	Source Material	Measurement Method (Probe)
N-doped carbon dots [205]	19	Coal	UV-Vis (DBPF)
N,S-codoped carbon dots [206]	11	Dansyl chloride	UV-Vis (DBPF)
CQD [207]	71	Riboflavin	Visible photoluminescence (SOSG)
GQD [63]	130	Polythiphene derivative	UV-Vis (Na-ADPA)
CQD [208]	27	Polythiphene benzoic acid	UV-Vis (Na-ADPA)
Mn/HA-CQD [209]	40	Manganese atoms and hyaluronic acid	UV-Vis (Na-ADPA)
N,S-codoped CQDs [210]	8	Polythiphene derivative	UV-Vis (Na-ADMA)
Sn@S-CQD [211]	37	Sodium *p*-styrene sulfonate and SnCl_4_	UV-Vis (ABDA)
CQD [212]	5.7	Trinitropyrene	Luminescence at 1270 nm
Cu-CQD [213]	36	Poly(acrilic acid) and Cu(NO_3_)_2_	UV-Vis (ABDA)
CQD [214]	62	pheophytin	UV-Vis (DBPF)
Mn-CD [215]	40	Manganese-phthalocyanine	UV-Vis (DBPF)
CQD [109]	33	PF 68 copolymer	Luminescence at 1270 nm

## Data Availability

Data sharing not applicable. No new data were created or analyzed in this study. Data sharing is not applicable to this article.
